# Supplementary material for: Unpicking the Secrets of African Swine Fever Viral Replication Sites
Source: Viruses. 2021 Jan 8;13(1):77. doi: 10.3390/v13010077 (PMC7827680; doi:10.3390/v13010077)
Supplement: Supplementary file 1 [file viruses-13-00077-s001.zip › Supplemental data/Supplemental data_Aicher.docx]

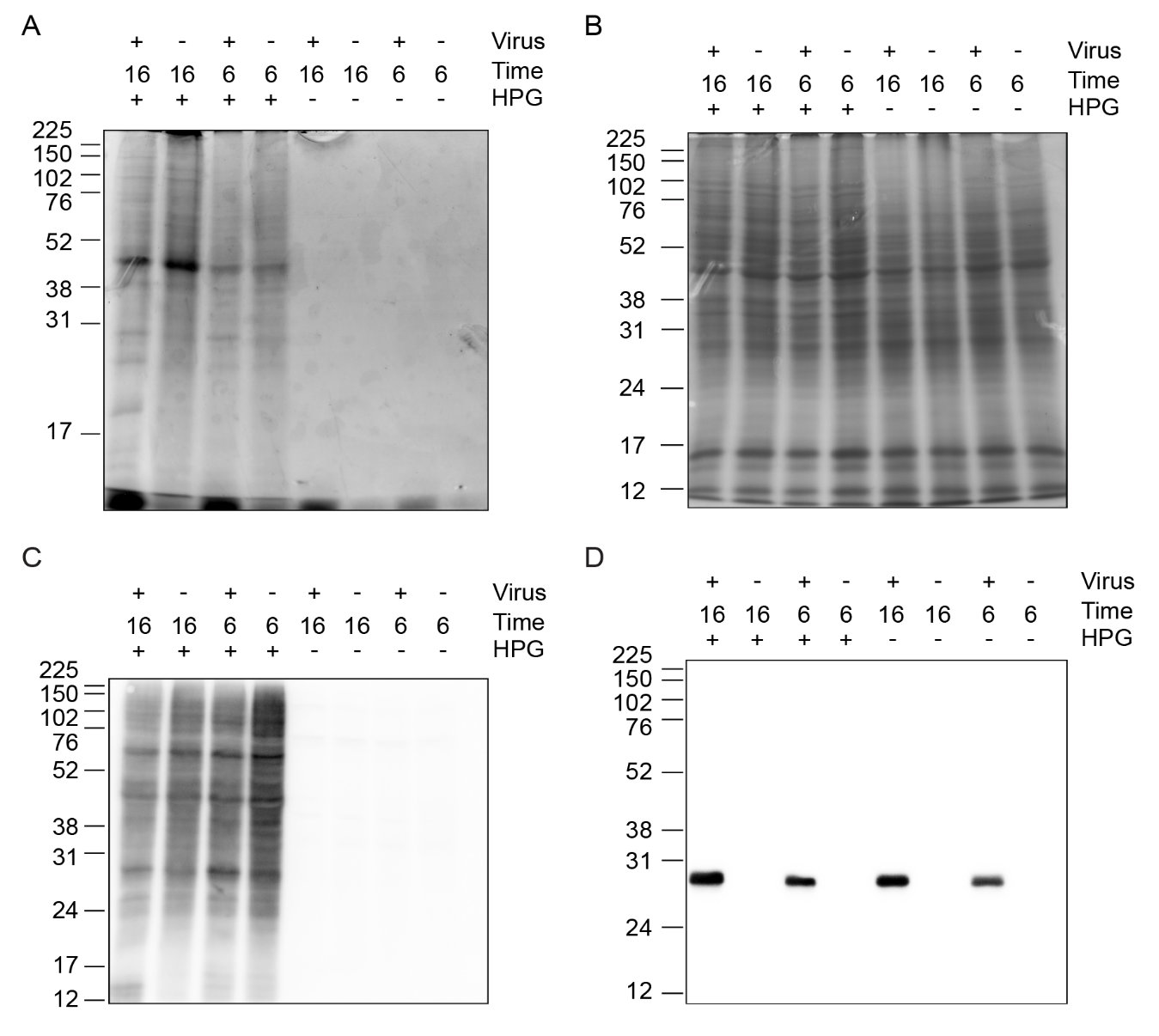


**Figure S1.** **Incorporation of HPG into proteins.** SDS-PAGE was used to confirm that the HPG signal seen in Fig. 3 represented incorporation into individual proteins. Whole cell lysates were prepared from ASFV or mock infected Vero cells pulsed at a final concentration of 1 mM with HPG or with standard DMEM for 1 h at 6 or 16 hpi. One hundred µg of each protein sample were subject to Click-IT reaction with azide conjugated to Alexa555 or biotin. Equivalent amounts of protein were then separated by electrophoresis and HPG-labelled proteins visualized by in-gel fluorescence (Fig. S1A) or immunoblotting (Fig. S1C). Distinct bands could be seen in cells labelled with HPG indicating incorporation of the methionine analogue into proteins, these could not be seen in cells that were not incubated with HPG showing that the signal was specific. Coomassie Blue staining was used to confirm loading of all lanes in the gel (Fig. S1B). Expression of viral proteins was confirmed by immunoblotting with an antibody against p30 an early ASFV protein (Fig. S1D) and there was no apparent difference in the intensity of the p30 signal between HPG labelled and unlabelled cells. Taken together these data showed that HPG was incorporated into nascent proteins (Fig S1) and could be used to visualise protein synthesis in cells (Fig 3, main text).


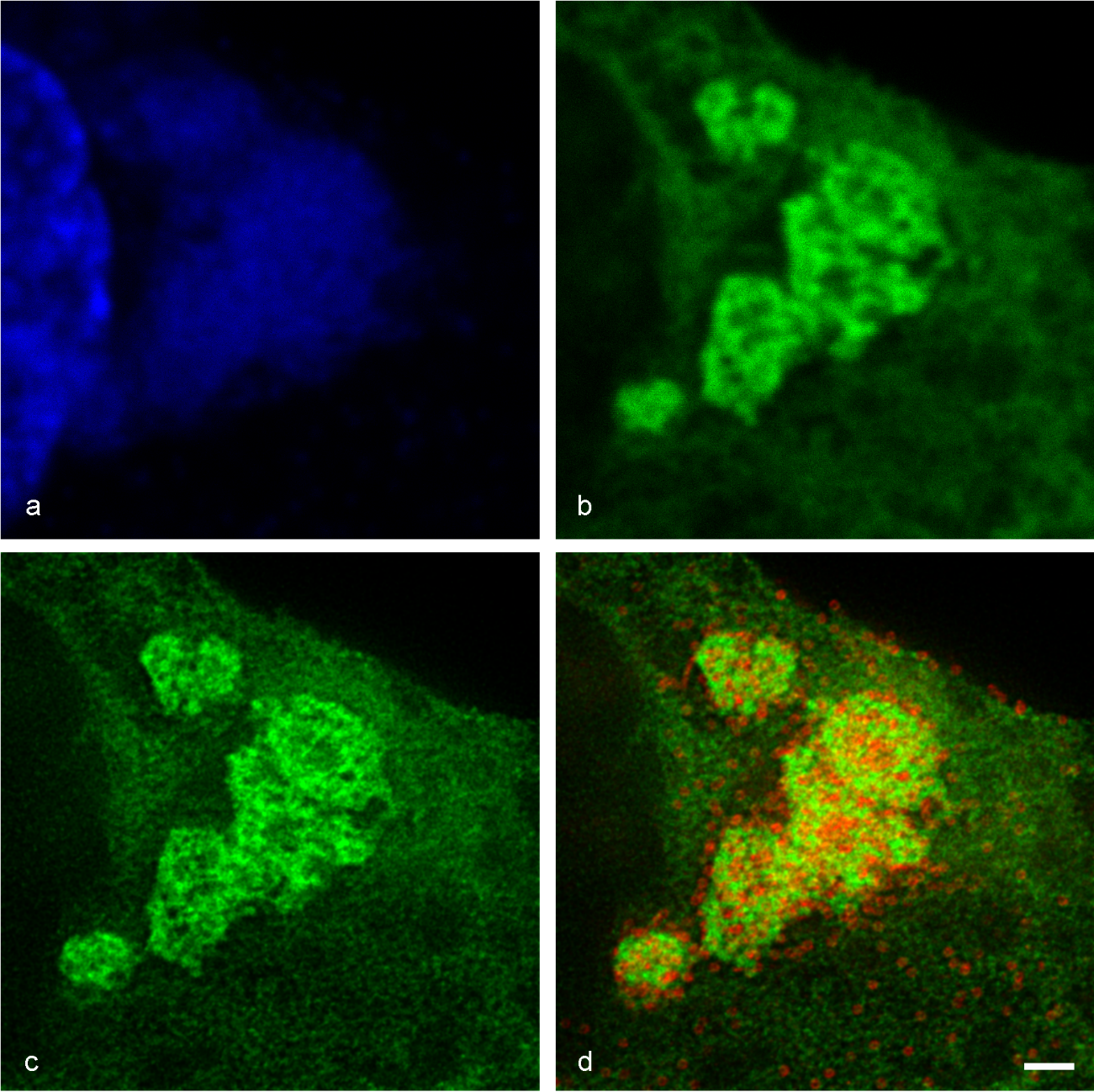


**Figure S2. Confocal and STED microscopy of a Vero cell infected with ASFV for 18 hours.** Viral DNA can be seen with ToPro3 staining (a) and an antibody against p34 highlights its location in the factory by confocal microscopy (b). STED imaging of the same factory reveals the sub-structure within the p34 ribbon as an intricate network (c) surrounded by new virions, here labelled with an antibody against p72. Scale = 1µm.


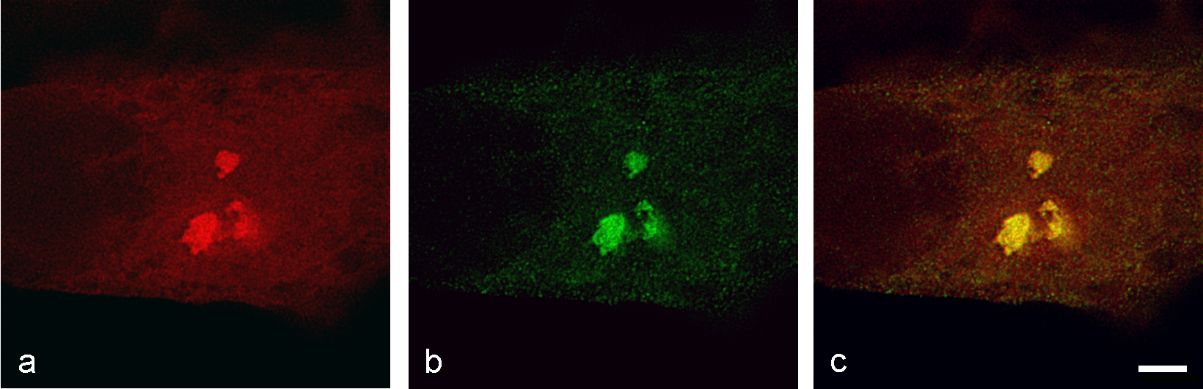


**Figure S3**. **STED microscopy of HPG incorporation into newly synthesized protein in the factory.** (a) HPG signal accumulates in the factory, (b) antibody against p34 ASFV protein reveals an intricate sub-structure to the protein mass, very similar to the pattern seen when imaging the antibody against p54 by STED (Fig 1, main text).

**Video S1. I. Tomographic reconstruction of HPF Vero cells at 18 hpi.**

**Video S2. II. Tomographic reconstruction of HPF Vero cells at 18 hpi**

**Video S3. III. Tomographic reconstruction of HPF Vero cells at 18 hpi.**

**Video S4. Early ASFV factory (12 hpi) seen by electron tomography.**
